# Effect of preconception multiple micronutrients vs. iron–folic acid supplementation on maternal and birth outcomes among women from developing countries: a systematic review and meta-analysis

**DOI:** 10.3389/fnut.2024.1390661

**Published:** 2024-06-14

**Authors:** Rashmi Ranjan Das, Jhuma Sankar, Nishant Jaiswal, Bhagirathi Dwibedi, Amit Kumar Satapathy, Pranita Pradhan, Prajyoti Sahu

**Affiliations:** ^1^Department of Pediatrics, AIIMS Bhubaneswar, Bhubaneswar, India; ^2^Department of Pediatrics, AIIMS New Delhi, New Delhi, India; ^3^School of Health and Wellbeing, University of Glasgow, Glasgow, United Kingdom; ^4^ICMR Advanced Centre for Evidence Based Child Health, PGIMER, Chandigarh, India

**Keywords:** multiple micronutrients, pre-conception, adolescent girls, pregnancy, maternal undernutrition, newborn

## Abstract

**Background:**

Maternal malnutrition affects the somatic growth of the fetus and subsequent adverse events during infancy and childhood period. Though trials have been conducted on multiple micronutrient (MMN) supplements initiated during the preconception period, there is no collated evidence on this.

**Materials and methods:**

We performed a systematic review of published trials with the application of Grading of Recommendations Assessment, Development, and Evaluation (GRADE). The searches were conducted until 30 September 2023. Meta-analysis was performed using Review Manager 5 software. The primary objective was to compare the effect of preconception MMN vs. iron–folic acid (IFA) supplementation on newborn anthropometric parameters at birth.

**Results:**

Of the 11,832 total citations retrieved, 12 studies with data from 11,391 participants [Intervention = 5,767; Control = 5,624] were included. For the primary outcome, there was no significant difference in the birth weight [MD, 35.61 (95% CI, −7.83 to 79.06), *p* = 0.11], birth length [MD, 0.19 (95% CI, −0.03 to 0.42), *p* = 0.09], and head circumference [MD, −0.25 (95% CI, −0.64 to −0.14), *p* = 0.22] between the MMN and control groups. For all the secondary outcomes [except for small for gestational age (SGA) and low birth weight (LBW)], the difference between the MMN and control groups was not significant. The GRADE evidence generated for all the outcomes varied from “very low to moderate certainty.”

**Conclusion:**

A “very low certainty” of evidence suggests that MMN supplementation may not be better than routine IFA supplementation in improving newborn anthropometric parameters (weight, length, and head circumference). The adverse events resulting from the supplementation were not significant. We need better quality uniformly designed RCTs before any firm recommendation can be made.

**Systematic review registration**: identifier (CRD42019144878: https://www.crd.york.ac.uk/prospero/#searchadvanced).

## Introduction

Maternal nutritional status before (preconception period) and during pregnancy is very important for the wellbeing of the mother and the baby. The prevalence of maternal malnutrition varies from 10 to 20% in most countries. Malnutrition in women acts as a risk factor for maternal mortality (contributing to 20%) and adverse pregnancy outcomes (such as obstructed labor, fetal deaths, or stillbirths), as well as neonatal outcomes (including preterm birth, low birth weight (LBW), small for gestational age (SGA), and birth asphyxia) (1). The roles of micronutrients such as folic acid (FA), iron, zinc, and calcium during pregnancy are already proven as they improve maternal and neonatal outcomes (1–3). In a Cochrane systematic review, MMN supplementation during pregnancy (20 trials and 141,849 women) led to a significant decrease in the number of low birth weight (LBW) [relative risk (RR) 0.88; 95% confidence interval (CI), (0.85–0.91)] and small for gestational age (SGA) [RR, 0.92; 95% CI, (0.88–0.97)] babies (3). The number of preterm babies was decreased, but the effect was not significant [RR, 0.95; 95% CI, (0.9–1.01)]. The authors concluded that the evidence may provide a basis to guide the replacement of iron–folic acid (IFA) with MMN supplementation for pregnancy in low- and middle-income countries (LMICs) (3).

Several factors affect the weight and micronutrient status during pregnancy, including food insecurity and birth spacing (2). For this reason, pre-pregnancy care should aim at achieving and sustaining optimal nutritional intake and body weight. In addition, ensuring early and adequate intake of micronutrients during the preconception period would provide added benefits, especially in cases of the pregnancy is unplanned (2, 3). In South Asia, malnutrition affects more than 38% of adolescent girls, and the decline in the past decade has not been optimal (4). The risk factors that have the potential to affect maternal and neonatal health can also exist during adolescence. Early marriage is common in some of the low- and middle-income countries (LMICs) where an adolescent girl has a high chance of getting pregnant, thereby posing an increased risk of adverse birth outcomes later on (5). This negatively affects the health of adolescent mothers and their offspring’s health in the future. Approximately 11% of all births are attributed to adolescent mothers of 15–19 years of age, and > 90% of these occur in LMICs.

MMNs (including vitamins and minerals) play a critical role in cellular metabolism, growth, and maintenance of normal functioning of the human body. MMN supplementation during pregnancy improves outcomes through placental function, including modulation of inflammation, oxidative stress, and vascular function (3). The isolated deficiency of these micronutrients rarely exists; as a result, it is difficult to assign a clinical or pre-clinical condition to the deficiency of a single micronutrient. Hence, MMN supplementation has been suggested as a cost-effective way to achieve multiple benefits. Providing appropriate interventions (e.g., MMN supplementation) during the preconception period as well as during pregnancy can be crucial to reducing adverse health outcomes. Keeping this in mind, micronutrient supplementation is currently being used as a strategy to improve nutrition in resource-poor settings (2).

Because of the adverse health risks associated with maternal anemia during pregnancy, iron and folic acid (IFA) supplementation is part of antenatal care (6, 7). However, recent systematic reviews have found the beneficial roles of multiple micronutrients (MMNs) over IFA when supplemented during pregnancy (8, 9). There have been published clinical trials that have investigated the efficacy of different MMN supplements during preconception on birth outcomes, and the results have been variable (10–21). The World Health Organization (WHO) in 2016 “did not recommend” MMN supplementation during pregnancy; however, in 2020, this was revised to “recommended in the context of rigorous research” (22). The reason was that while the evidence suggests that there may be a limited benefit and little harm in replacing iron and folic acid supplements with MMN, the evidence on low birth weight and its component parts (preterm birth and SGA) is difficult to interpret. Keeping this in mind and in light of the availability of new data, the present review was conducted to update the knowledge and provide evidence for the formulation of future guidelines on MMN supplementation during the peri-conceptional period.

## Materials and methods

The review protocol was registered at PROSPERO with registration number: CRD42019144878.

### Criteria for considering studies for this review

#### Types of studies

Randomized controlled trials (including cluster RCTs).

#### Types of participants

Married women aged 15–45 years who are either nulliparous or multiparous (parity 0–5), with no current or planned contraceptive use, and who became pregnant ≥3 months after supplementation were included. Women with a history of obstetric complications, those not willing for hospital delivery, and those with uncorrected anemia (hemoglobin ≤8 g/dL) were excluded.

#### Types of interventions

The MMN supplements used within the intervention arm are consistent with the UNICEF/WHO/UN International Multiple Micronutrient Preparation (UNIMMAP). The intervention has to be supplied (must) during the preconception period but is optional during pregnancy. During pregnancy, IFA supplementation has to be provided as per the WHO recommendation.

#### Types of control

No supplementation or only folic acid (during preconception prevention of neural tube defect) during the preconception period and IFA supplementation during pregnancy have to be provided as per the WHO recommendation.

### Types of outcome measures

#### Primary outcomes

- Birth weight, length, and head circumference for gestational age of the newborn (measured within 48 h of birth).

#### Secondary outcomes

- Maternal weight gain (measured at baseline, then monthly throughout pregnancy or at least once during the first and third trimesters, during the first week postpartum, and finally at 3 and 6 months postpartum).
- Adverse pregnancy outcomes (monitored during monthly check-ups or at any time during pregnancy, and within the first week postpartum).
- Adverse newborn outcomes (monitored at birth, within the first week postpartum, and at 28 weeks of age).
- Adverse events resulting from supplementation (monitored throughout supplementation until the first week postpartum).
- Long-term growth outcome (weight, height, and head circumference) in the offspring (measured at 3, 6, 9, and 12 months of age).
- Long-term neurodevelopmental outcome in the offspring (measured at 3, 6, 9, 12, 18, and 24 months of age).
- Postpartum maternal cognition, depression, and caregiving (assessed within the first week postpartum and then at 3 and 6 months postpartum).

### Search methods for identification of studies

We conducted a comprehensive search, including the Cochrane Central Register of Controlled Trials (CENTRAL) in the Cochrane Library; MEDLINE via PubMed (1980 to 30 September 2023); and Embase (1980 to 30 September 2023) using each PICO (patient/population, intervention, comparison and outcomes) term. The details of the search strategy have been provided in Supplementary Table S1. We did not apply language restrictions. We searched clinical trial registries for ongoing and recently completed clinical trials.^1^

We also searched for abstracts from key nutritional annual meetings. We strived for additional citations by using the references in the articles retrieved through the searches. However, we did not contact subject experts to identify unpublished and ongoing studies (as pre-specified in the protocol), as some of the studies were already in the clinical trial registry.

### Screening and data collection

Two authors independently screened the titles and abstracts of articles identified by searches for eligibility. Data extraction from each included study was carried out using a pre-designed data extraction form. Disagreements were resolved through discussion with a third author.

### Assessment of risk of bias in included studies

We used the ‘Risk of bias’ assessment tool and the criteria set out in the *Cochrane Handbook for Systematic Reviews of Interventions* to assess the risk of bias for included studies (23). We also looked for sources of bias originating from differences between individual RCTs and cluster RCTs (e.g., the relationship between allocation concealment and recruitment bias may be greater in cluster RCTs). Two authors independently assessed the risk of bias in the included studies, and any disagreement was resolved through discussion with a third author.

### Assessment of reporting (publication) bias

We assessed reporting biases by trying to identify whether the study was included in a trial registry, whether a protocol is available, and whether the Methods section provides a list of outcomes. We compared the list of outcomes from those sources vs. the outcomes reported in the published article. The inverted funnel was constructed to check for possible publication bias.

### Measures of treatment effect

We performed statistical analysis according to statistical guidelines referenced in the *Cochrane Handbook for Systematic Reviews of Interventions* (23). For dichotomous outcomes, we expressed measures of effects as typical risk ratios (RRs) and typical risk differences (RDs) with 95% confidence intervals (CIs). For continuous outcomes, we expressed measures of effect as weighted mean differences (MDs) with 95% CIs. We used the generic inverse variance method in Review Manager 5 to perform a meta-analysis using inflated variances (24). Considering the types of MMN supplements (different preparations and different schedules) as a random factor, a random-effects model was used for all the analyses. We assessed statistical heterogeneity via visual inspection of forest plots of included trials, using the chi-square test and the I^2^ statistic. We used the following cutoffs for the results of the I^2^ test: < 50% low, 50 to 74% moderate, and ≥ 75% high heterogeneity. We attempted to identify the reason for heterogeneity by conducting either subgroup analyses or sensitivity analyses.

### Certainty of evidence

We used the GRADE approach, as outlined in the GRADE Handbook, to assess the certainty of evidence (25). We used GRADEproGDT (*GRADEpro 2016*) to create a ‘Summary of findings’ table to report the certainty of evidence (26).

### Subgroup analysis and investigation of heterogeneity

We conducted subgroup analyses to explore heterogeneity. We analyzed the effects of MMN intervention in the following subgroups.

- Types of MMN supplementation (tablet, capsule, *or* sachet form; lipid vs. non-lipid-based formulations)
- Types of RCTs (one-stage vs. two-stage randomization)

### Sensitivity analysis

We conducted sensitivity analyses to assess the impact of a high risk of bias on the outcome of meta-analyses by adding studies with a high risk of bias to pooled studies with a low risk of bias. For completeness of sensitivity analysis, we also employed the leave-out one trial sensitivity analysis method to check the robustness of our meta-analysis for primary outcomes. The steps are as follows: (1) remove the first of the K studies and conduct the meta-analysis on the remaining K-1 studies; (2) remove the second of the K studies and conduct the meta-analysis on the remaining K-1 studies; (3) continue this process until there are K distinct meta-analyses (each with K-1 studies).

If the results of the K meta-analyses in the leave-one-out method are consistent, then there is confidence that the overall meta-analysis is robust.

## Results

### Description of studies

**Figure 1:**
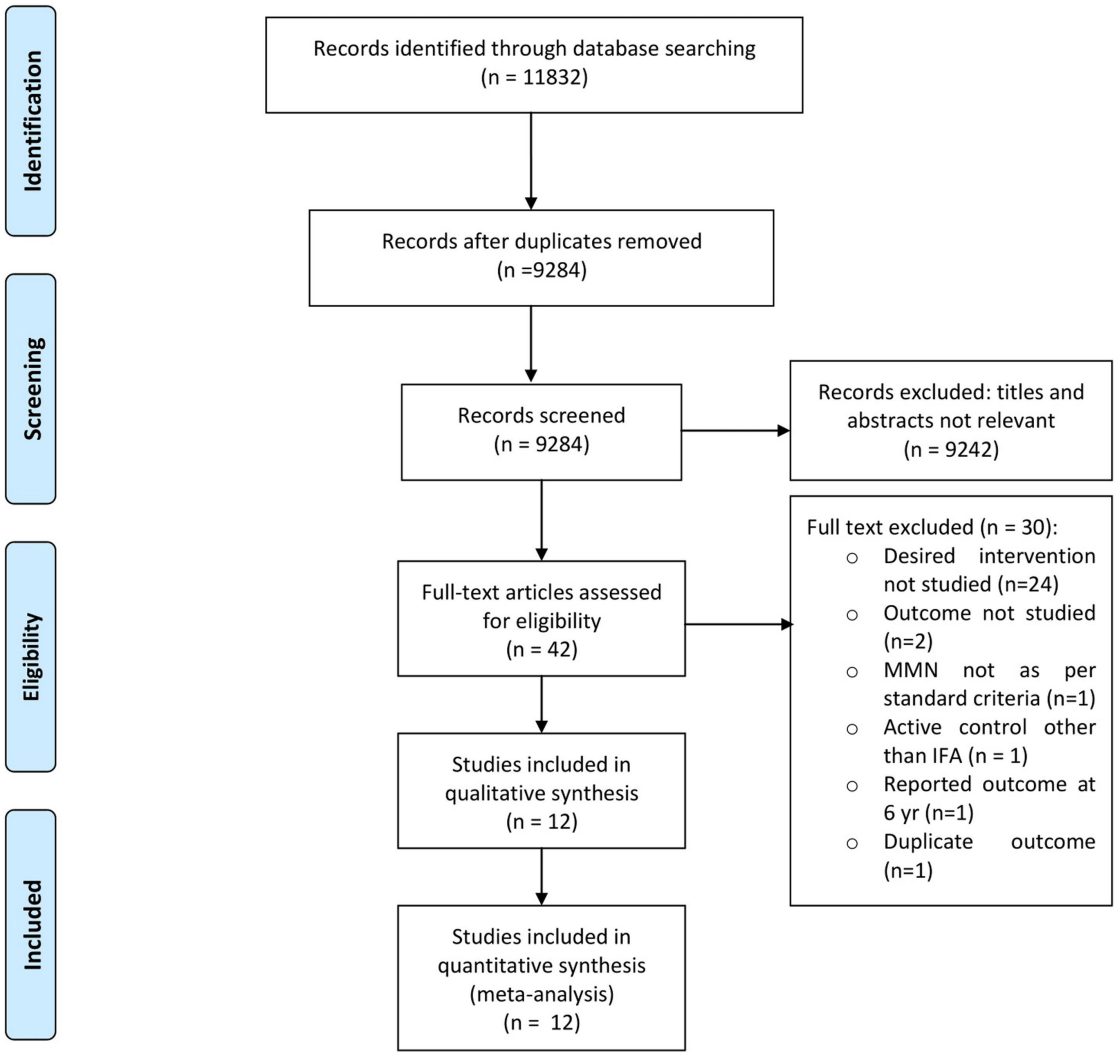
PRISMA flow diagram.

Of the 11,832 total citations retrieved, the full text of 42 articles was assessed for eligibility, and 30 were excluded for various reasons (Figure 1). The reasons were as follows: preconception supplementation of intervention was not studied (not part of ante-natal supplementation) [*n* = 24], outcomes of interest not studied [*n* = 2], MMN not as per standard (UNIMMAP) criteria and outcome of interest not studied [*n* = 1], active control other than IFA [*n* = 1], reported outcome at 6 years [*n* = 1], and duplicate outcome reported [*n* = 1]. Hence, finally, 12 studies [factorial design = 1] with data from 11,391 participants [intervention = 5,824; control = 5,680] were included (10–21).

The studies were conducted in the following countries: Vietnam, India, Pakistan, Congo, Guatemala, Gambia, and Indonesia. One study was not registered in any clinical trial registry (20). Another study was registered with the Thai Clinical Trial Registry (TCTR), but we could not retrieve the details (10). In three studies, the supplementation started approximately 3 months or so before conception (17–19). In the remaining nine studies, the duration of supplementation was variable before conception (10–16, 20, 21). In all the studies, MMN supplementation was stopped once pregnancy was confirmed, and IFA was continued until delivery. In one study (4 arm) intervention, the age range of participants varied from 16 to 45 years, and all were non-pregnant women. Two studies used lipid-based supplementations as MMN sources (16, 17). Two studies used sachet preparation of MMN (16, 17), two used tablet preparation (11, 21), and the remaining used MMN in capsule form. Table 1 describes other characteristics of the included studies.

**Table 1:** Characteristics of included studies.

Study author [Reference]	Year of study, Country	Study design, setting	Sample size (*N*), age of participants	Intervention group (dose schedule)	Duration of intervention	Standard care (control) group	Additional comments
Sumarmi et al. (10)	2011–2012; Indonesia	RCT (2 arm); Community setting	Randomized: 420 (intervention = 210, control = 210) Age: 16–35 years	Dark-green leafy vegetables and animal source foods were used to prepare capsules that were given on an alternate day	Variable	Iron–folic acid	Studied the effect of MMN on cord blood IGF-1 level. Funded study.
Owens et al. (11)	2006–2008; Gambia	RCT (2 arm); Community setting	Randomized: 3206 (*1156; intervention = 567, control = 589) Age: 17–45 years	Tablets of MMN provided daily	Variable	Iron–folic acid	Studied the effect of MMN on placental function. Funded study.
Ramakrishnan et al. (12)	2011–2014; Vietnam	PRECONCEPT study: RCT (3 arm); Community setting	Randomized: 5011 (*1040; intervention = 525, control = 515) Age: 18–40 years	Capsule of MMN provided weekly	1–26 months	Iron–folic acid	Maternal mental health during pregnancy and postpartum from PRECONCEPT study was analyzed. Compliance was 90%. Funded study.
Nguyen et al. (13)	2011–2014; Vietnam	PRECONCEPT study: RCT (3 arm); Community setting	Randomized: 5011 (*1026; intervention = 508, control = 518) Age: 18–40 years	Capsule of MMN provided weekly	1–26 months	Iron–folic acid	Women consumed supplements ≥26 weeks before conception. The study looked at anemia and iron status only. Funded study.
Nguyen et al. (14)	2011–2014; Vietnam	PRECONCEPT study: RCT (3 arm); Community setting	Randomized: 5011 (*955; Intervention = 478, Control = 477) Age: 18–40 years	Capsule of MMN provided weekly	1–26 months	Iron–folic acid	2-year follow-up data of PRECONCEPT study. Compliance was 90%. The maximum duration of preconception intervention was 2 y. Funded study.
Nguyen et al. (15)	2011–2014; Vietnam	PRECONCEPT study: RCT (3 arm); Community setting	Randomized: 5011 (*1044; intervention = 518, control = 526) Age: 18–40 years	Capsule of MMN provided weekly	1–26 months	Iron–folic acid	Maternal mental health during pregnancy and postpartum from the PRECONCEPT study was analyzed. Compliance was 90%. The maximum duration of preconception intervention was 2 y. Funded study.
Hambidge et al. (16)	2013–2017; multi-country (India, Pakistan, Congo, Guatemala)	Women First Trial: cluster RCT (3 arm); Community setting	Randomized: 7387 (*2124; intervention = 1,029, control = 1,095) Age: 16–35 years	Lipid-based supplementation; One sachet daily	3 months	Iron–folic acid	The supplement provided 2.6 g protein and 118 kcal. Compliance with supplement was >87%. Funded study.
Dhaded et al. (17)	2013–2017; multi-country (India, Pakistan)	Women First Trial: cluster RCT (3 arm); Community setting	Randomized: 3836 (*632; intervention = 333, control = 299) Age: 16–35 years	Lipid-based supplementation; One sachet daily	3 months	Iron–folic acid	Secondary analysis of Women First trial. An additional protein-energy supplement was provided to women whose BMI was <20 kg/m2. Modified ITT analysis done. Funded study.
Nga et al. (18)	2011–2015; Vietnam	VINAVAC study: RCT (3 arm); Community setting	Randomized: 460 (*307; intervention = 150, control = 157) Age: 18–30 years	Dark-green leafy vegetables and animal source foods were used to prepare capsules that were given 5 days a week	2–3 months	Standard peri-natal care	The supplement provided at least 50% of a pregnant woman’s recommended dietary allowance for five nutrients: iron, zinc, folate, vitamin A, and vitamin B12. High attrition rate (31%). Modified ITT analysis done. Funded study.
Quyen et al. (19)	2011–2015; Vietnam	VINAVAC study: RCT (3 arm); Community setting	Randomized: 460 (*207; intervention = 101, control = 106) Age: 18–30 years	Dark-green leafy vegetables and animal source foods were used to prepare capsules that were given 5 days a week	11 months	Standard peri-natal care	The supplement provided at least 50% of a pregnant woman’s recommended dietary allowance for five nutrients: iron, zinc, folate, vitamin A, and vitamin B12. High attrition rate (31%). Modified ITT analysis done. Funded study.
Widasari et al. (20)	2016–2018; Indonesia	RCT (2 arm); Community setting	Randomized: 19 (intervention = 12, control = 7) Age: 18–35 years	MMN was provided weekly through capsule	Variable	Iron–folic acid	MMN was provided daily during pregnancy. Funded study.
Taneja et al. (21)	2017–2019; India	RCT (4 arm); factorial design, individually randomized	Randomized: 2461 (intervention = 1,326, control = 1,135) Age: 18–30 years	MMN was provided thrice weekly through tablets	4–6 months (median)	Weekly IFA to those without anemia	Open-label trial. Extra calories and protein were given to women with under-nutrition. WaSH (water, sanitation, and hygiene) intervention was also provided. The trial provided additional data on supplementation during pre-conceptio+pregnancy+childhood.

^*^Data of participants included in the present systematic review. ITT, Intention to treat; MMN, multiple micronutrients; IFA, iron–folic acid; IGF-1, insulin growth factor-1.

### Risk of bias in included studies

The details are provided in the Supplementary Figure S1. Random sequence generation was unclear in one study (20). Five studies were open-label (16–19, 21). Six studies reported higher attrition rates (11–16). Three studies were found to report the outcomes selectively (10, 12, 20). Overall, the included studies were assessed as having a low to moderate risk of bias.

### Effect of interventions

#### Primary outcomes

**Figure 2:**
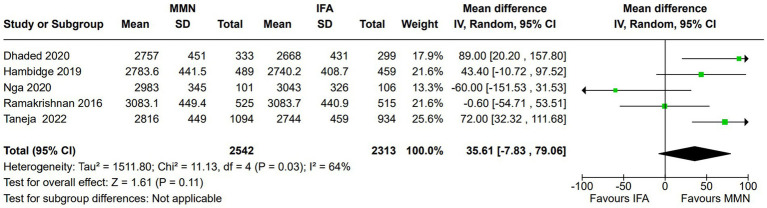
Forest plot showing birth weight.

**Figure 3:**
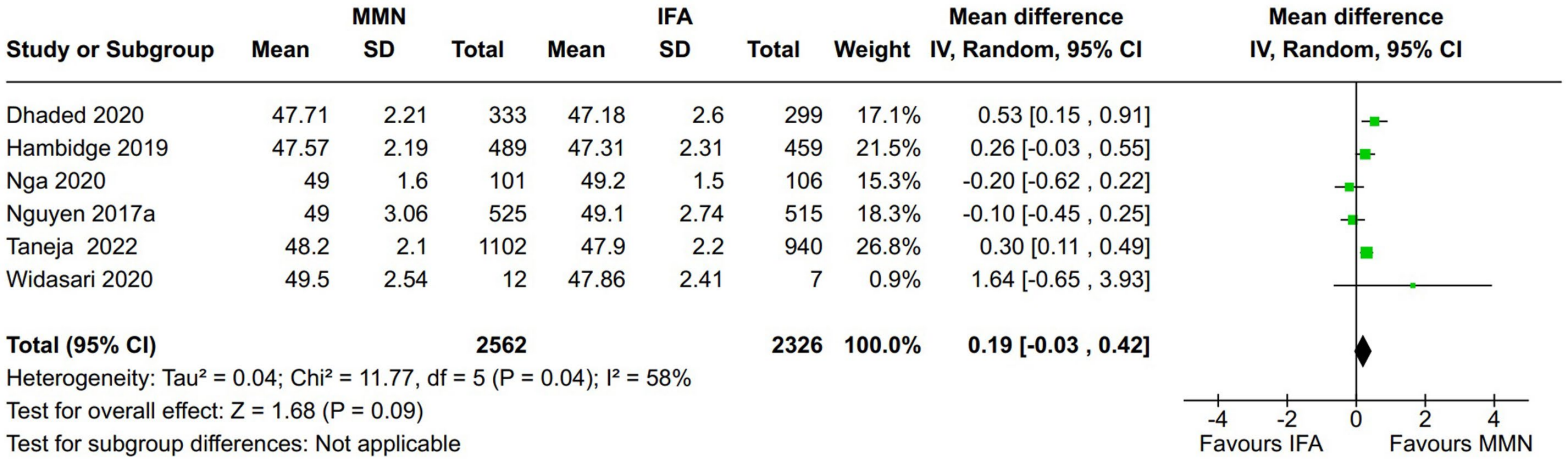
Forest plot showing birth length.

**Figure 4:**
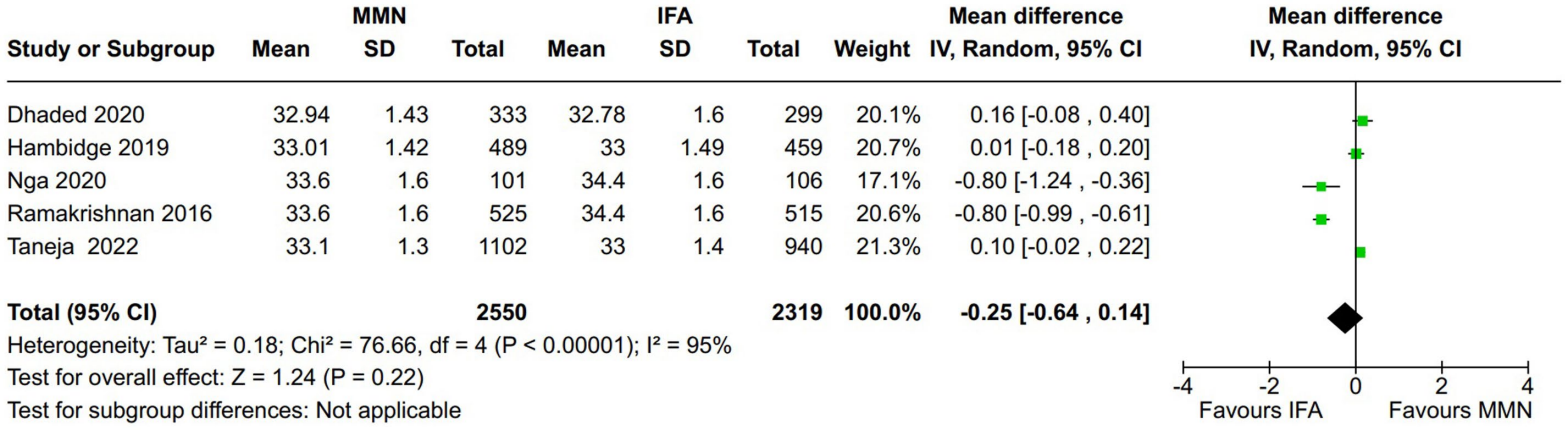
Forest plot showing the head circumference.

- Birth weight (g):

- Five studies reported this outcome (12, 16–18, 21). Two studies provided MMNs as lipid-based formulations (16, 17), and the remaining three studies provided them as non-lipid-based formulations in capsule/tablet form. Data from 4,855 participants were included in the analysis. There was no significant difference in the birth weight (g) between the MMN and control groups [MD, 35.61 (95% CI, −7.83 to 79.06), *p* = 0.11] (Figure 2). The heterogeneity was significant [I^2^ = 64%].
- Subgroup analysis: Two studies (1,580 participants) providing lipid-based MMN supplementation in sachet form found a significant difference in birth weight between the MMN and control groups [MD, 58.77 (95% CI, 14.7 to 102.84), *p* = 0.009] with insignificant heterogeneity (I^2^ = 0%) (16, 17). One study (2028 participants) that adopted two-stage randomization found a significant difference in the birth weight between the MMN and control groups [MD, 72 (95% CI, 32.32 to 111.68), *p* = 0.0004] (21).
- Sensitivity analysis: In the leave-out trial analysis, we found that removing one trial (18) led to a significant difference in birth weight between the MMN and control groups [MD, 50.33 (95% CI, 13.52 to 87.13), *p* = 0.11] without significant heterogeneity [I^2^ = 48%].

- Birth length (cm):

- Six studies reported this outcome (15–18, 20, 21). Two studies provided MMNs as lipid-based formulations (16, 17), and the remaining four studies provided them as non-lipid-based formulations in capsule/tablet form. Data from 4,888 participants were included in the analysis. There was no significant difference in the birth length (cm) between the MMN and control groups [MD, 0.19 (95% CI, −0.03 to 0.42), *p* = 0.09] (Figure 3). The heterogeneity was significant [I^2^ = 58%].
- Subgroup analysis: Two studies (1,580 participants) providing lipid-based MMN supplementation in sachet form found a significant difference in birth length between the MMN and control groups [MD, 0.36 (95% CI, 0.13 to 0.59), *p* = 0.002] with insignificant heterogeneity [I^2^ = 19%] (16, 17). One study (2042 participants) that adopted two-stage randomization found a significant difference in the birth length between the MMN and control groups [MD, 0.3 (95% CI, 0.11 to 0.49), *p* = 0.002] (21).
- Sensitivity analysis: In the leave-out trial analysis, we found that removing two trials (14, 18) led to a significant difference in birth length between the MMN and control groups without significant heterogeneity. When one trial was removed (14), the results from the remaining five trials were as follows: [MD, 0.26 (95% CI, 0.03 to 0.49), *p* = 0.03, I^2^ = 50%], and when the other trial was removed (18), the results from remaining five trials were as follows: [MD, 0.26 (95% CI, 0.05 to 0.48), *p* = 0.03, I^2^ = 47%].

- Head circumference (cm):
- Five studies reported this outcome (12, 16–18, 21). Two studies provided MMNs as lipid-based formulations (16, 17), and the remaining four studies provided them as non-lipid-based formulations in capsules/tablet form. Data from 4,869 participants were included in the analysis. There was no significant difference in the head circumference (cm) between the MMN and control groups [MD, −0.25 (95% CI, −0.64 to 0.14), *p* = 0.22] (Figure 4). The heterogeneity was significant [I^2^ = 95%].
- Subgroup analysis: No significant difference was found in head circumference between the MMN and control groups in different supplement groups (lipid-based sachets or non-lipid-based capsules/tablets). One study that adopted two-stage randomization did not find a significant difference in the head circumference between the MMN and control groups (21).
- Sensitivity analysis: In the leave-out trial analysis, no difference was found (the results remained not significant, and the heterogeneity remained high).

#### Secondary outcomes

**Figure 5:**
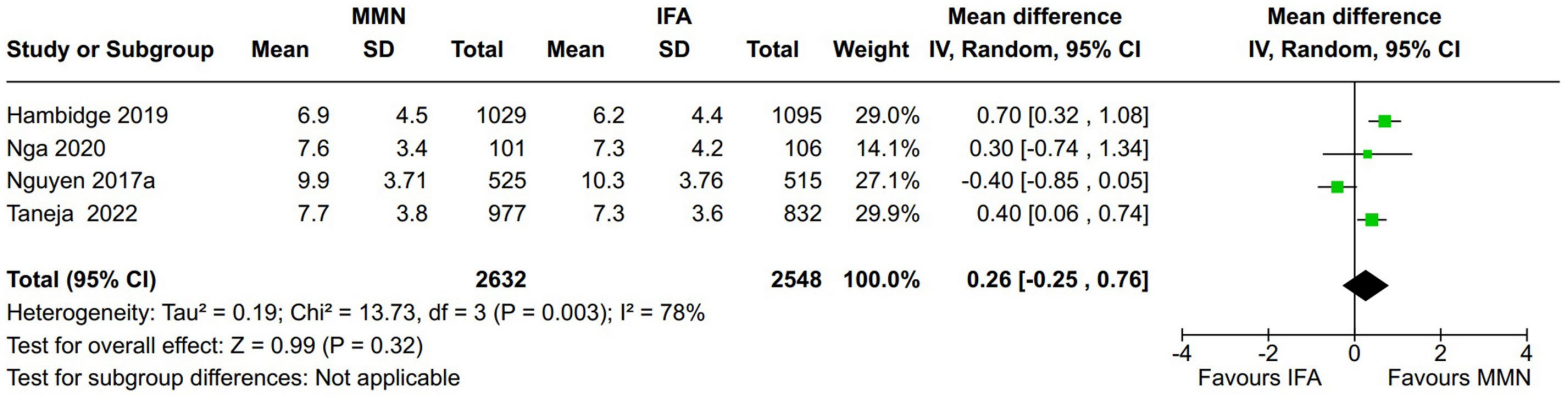
Forest plot showing maternal weight gain.

**Figure 6:**
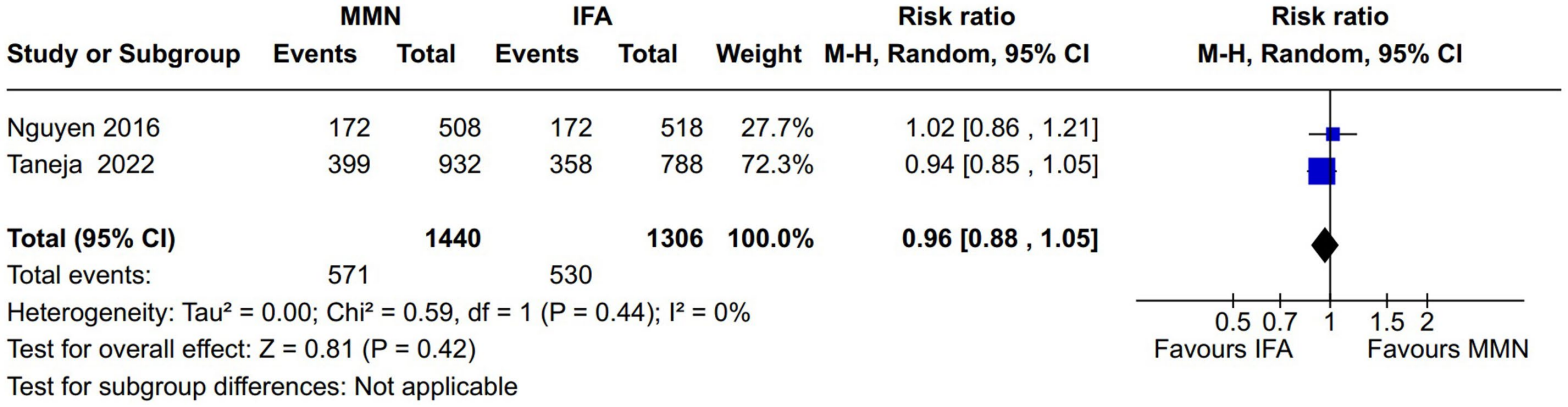
Forest plot showing maternal anemia.

**Figure 7:**
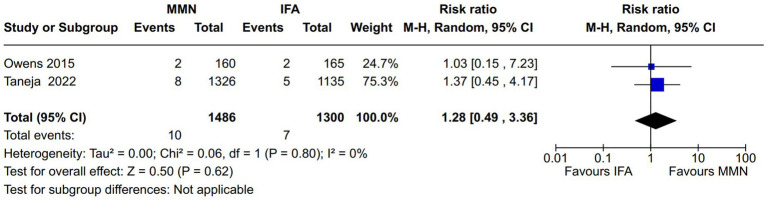
Forest plot showing maternal death.

**Figure 8:**
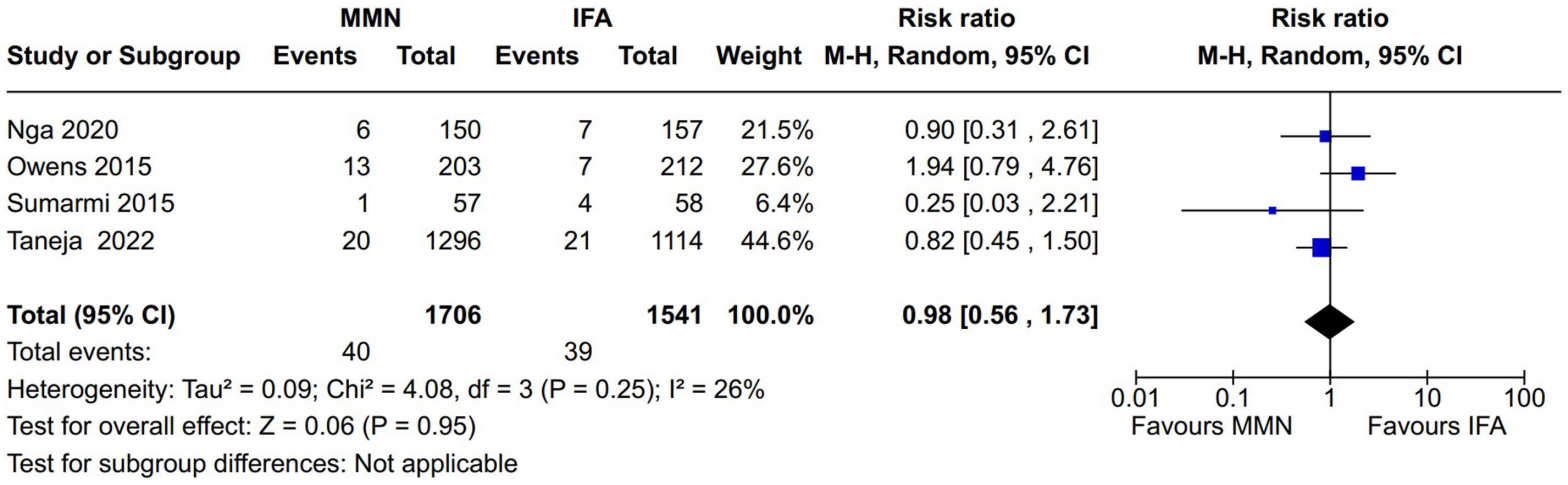
Forest plot showing fetal death.

**Figure 9:**
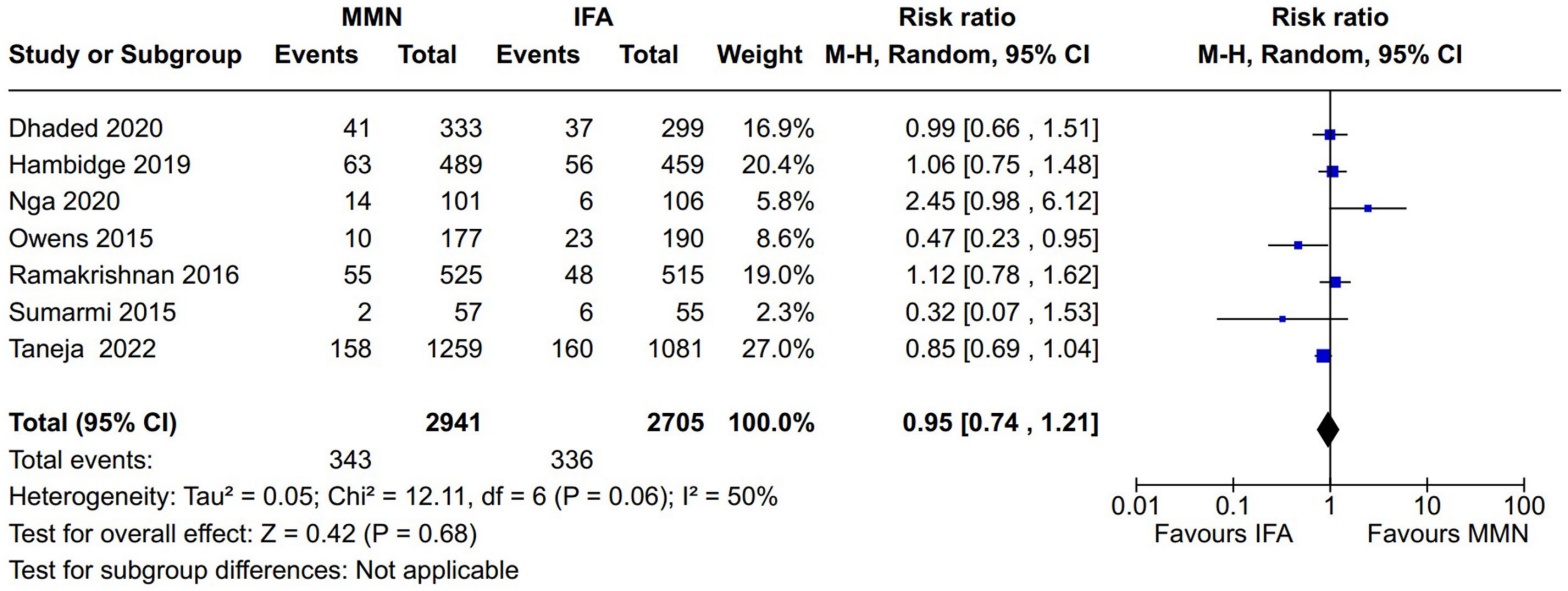
Forest plot showing pre-term delivery.

**Figure 10:**
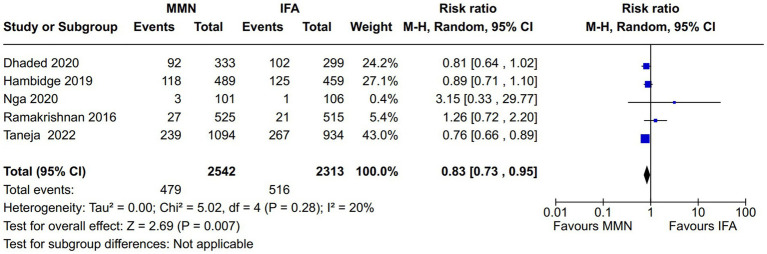
Forest plot showing low birth weight.

**Figure 11:**
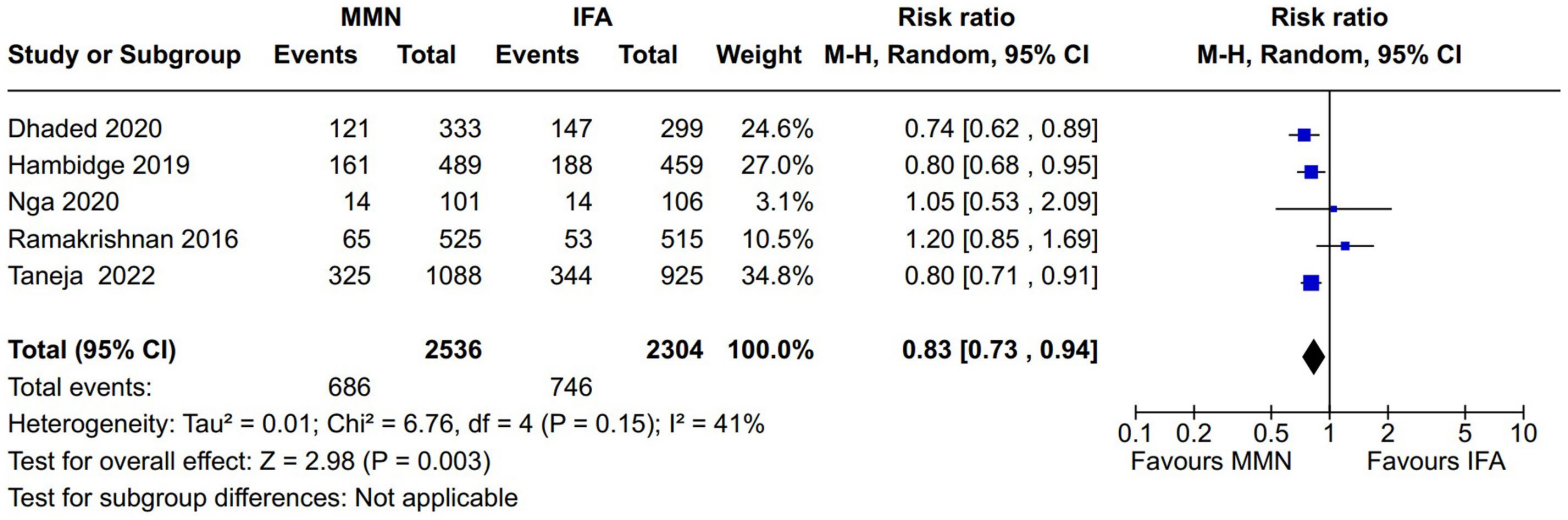
Forest plot showing small for gestational age.

**Figure 12:**
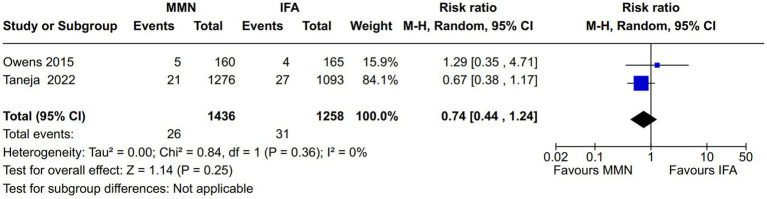
Forest plot showing neonatal death.

- Maternal weight gain: Four studies reported this outcome during pregnancy (15, 16, 18, 21). One study provided MMNs as lipid-based supplementation (16), and the other provided them as non-lipid-based formulations through capsules/tablets. Data from 5,180 participants were included in the analysis. There was no significant difference in the weight gain (kg) between the MMN and control groups [MD, 0.26 (95% CI, −0.25 to 0.76), *p* = 0.32] (Figure 5). The heterogeneity was significant [I^2^ = 78%].
- Maternal anemia: Two studies reported this outcome (13, 21). Both provided MMNs as non-lipid-based formulations through capsules/tablets. Data from 2,746 participants were included in the analysis. There was no significant difference in the maternal anemia rate between the MMN and control groups [RR, 0.96 (95% CI, 0.88 to 1.05), *p* = 0.42] (Figure 6). There was no heterogeneity [I^2^ = 0%].
- Adverse pregnancy outcomes:

- Maternal death: Two studies reported this outcome (11, 21). Both provided MMNs as non-lipid-based formulations through tablets. Data from 2,786 participants were included in the analysis. There was no significant difference in the maternal death rate between the MMN and control groups [RR, 1.28 (95% CI, 0.49 to 3.36), *p* = 0.62] (Figure 7). There was no heterogeneity [I^2^ = 0%].
- Fetal death (miscarriages and stillbirth): Four studies reported this outcome (10, 11, 18, 21). The studies provided MMNs as non-lipid-based formulations in capsule/tablet form. There was no significant difference in the fetal death rate between the MMN and control groups [RR, 0.98 (95% CI, 0.56 to 1.73), *p* = 0.95] (Figure 8). The heterogeneity was not significant [I^2^ = 26%].

- Adverse newborn outcomes:
  Preterm delivery: Seven studies reported this outcome (10–12, 16–18, 21) (Figure 9). Two studies provided MMNs as lipid-based (16, 17), and the remaining five studies provided them in capsule/tablet form. Data from 5,646 participants were included in the analysis. There was no difference between the MMN and control groups [RR, 0.95 (95% CI, 0.74 to 1.21), *p* = 0.68]. The heterogeneity was not significant [I^2^ = 50%].
  Low birth weight (LBW): Five studies reported this outcome (12, 16–18, 21) (Figure 10). Two studies provided MMNs as lipid-based (16, 17), and the remaining three studies provided them in capsule/tablet form. Data from 4,855 participants were included in the analysis. There was a significant decrease in the rate of LBW in the MMN group compared to the control group [RR, 0.83 (95% CI, 0.73 to 0.95), *p* = 0.007]. The heterogeneity was not significant [I^2^ = 20%].
  Small for gestational age (SGA): Five studies reported this outcome (12, 16–18, 21) (Figure 11). Two studies provided MMN as lipid-based (16, 17), and the remaining three studies provided them in capsule/tablet form. Data from 4,840 participants were included in the analysis. There was a significant decrease in the rate of SGA in the MMN group compared to the control group [RR, 0.83 (95% CI, 0.73 to 0.94), *p* = 0.003]. The heterogeneity was not significant [I^2^ = 41%].
  Neonatal death: Two studies reported this outcome (11, 21) (Figure 12). These studies provided MMNs as tablets. Data from 2,694 participants were included in the analysis. There was no difference between the MMN and control groups [RR, 0.74 (95% CI, 0.44 to 1.24), *p* = 0.25]. There was no heterogeneity [I^2^ = 0%].
- Adverse events resulting from supplementation: None of the included studies reported any difference in the overall and individual adverse events resulting from the MMN supplementation.
- Long-term growth outcome: Two studies reported on changes in weight (wasting or underweight), length (stunting), and head circumference (cm) at various time points (6 mo, 12 mo, 18 mo, and 24 mo) (19, 21). These studies provided MMNs in capsule/tablet form. There was no difference in any of the outcomes at respective time points.
- Long-term neurodevelopmental outcome in the offspring: This was reported in one study (14). There was no difference between the MMN and control groups in any of the outcomes (motor, cognitive, and language).
- Postpartum maternal cognition, depression, and caregiving: One study reported on maternal depression (15). This study provided MMNs in capsule form. Data from 1,044 participants were included in the analysis. There was a significant difference neither in the depression score [MD, 0.09 (95% CI, −0.12 to 0.3), *p* = 0.41] nor in the proportions of women with postpartum depression [RR, 1.37 (95% CI, 0.74 to 2.54), *p* = 0.31] between the MMN and control groups.

### Publication bias

We constructed funnel plots for all three domains (birth weight, length, and head circumference) of the primary outcome (anthropometry at birth) to assess for publication bias. Asymmetry in the funnel plot was noted for the birth weight outcome, for which sensitivity analysis was conducted, but the result did not change (Supplementary Figure S2).

### Grade of evidence

The evidence generated was of “very low certainty” for all the primary outcomes (newborn anthropometric parameters—birth weight, length, and head circumference). For the secondary outcomes, the evidence generated was of “very low certainty” for maternal weight gain and maternal and fetal death; “low certainty” for preterm delivery and neonatal death; and “moderate certainty” for LBW, SGA, and maternal anemia. A detailed analysis of the summary of evidence is provided in Table 2.

**Table 2:** Preconception MMN supplementation vs. IFA for women of reproductive age.

Outcome [No. of participants (*N*), No. of studies]	Mean or Relative effect (95% CI)	Anticipated absolute effects (95% CI)			Certainty of evidence
		MMN	IFA	Difference	
Birth weight [N: 4855; 5 RCTs]	MD 35.61 (−7.83 to79.06)	Mean birth weight: 2855.78	Mean birth weight: 2840.54	35.61 more (7.83 fewer to 79.06 more)	⨁◯◯◯ Very low^a,b,c^
Birth length [N: 4888; 6 RCTs]	MD 0.19 (−0.03 to 0.42)	Mean birth length: 48.5	Mean birth length: 48.09	0.19 more (0.03 fewer to 0.42 more)	⨁◯◯◯ Very low^c,d,e^
Head circumference [N: 4869; 5 RCTs]	MD -0.25 (−0.64 to 0.14)	Mean head circumference: 33.25	Mean head circumference: 33.52	0.25 lower (0.64 lower to 0.14 higher)	⨁◯◯◯ Very low^a,d,e^
Maternal weight gain [N: 5180; 4 RCTs]	MD 0.26 (−0.25 to 0.76)	Mean maternal weight gain: 8.03	Mean maternal weight gain: 7.78	0.26 more (0.25 fewer to 0.76 more)	⨁◯◯◯ Very low^b,e,f^
Maternal death [N: 2786; 2 RCTs]	RR 1.28 (0.49 to 3.36)	0.5%	0.7% (0.3 to 1.8)	0.2% more (0.3 fewer to 1.3 more)	⨁◯◯◯ Very low^f,g^
Fetal death [N: 3247; 4 RCTs]	RR 0.98 (0.56 to 1.73)	2.5%	2.5% (1.5 to 3.9)	0.1% fewer (0.8 fewer to 1.3 more)	⨁◯◯◯ Very low^c,e,h^
Preterm delivery [N: 5646; 7 RCTs]	RR 0.95 (0.74 to 1.21)	12.4%	11.8% (9.2 to 15)	0.6% fewer (3.2 fewer to 2.6 more)	⨁⨁◯◯ Low^c,i^
Neonatal death [N: 2694; 2 RCTs]	RR 0.74 (0.44 to 1.24)	2.5%	1.8% (1.1 to 3.1)	0.6% fewer (1.4 fewer to 0.6 more)	⨁⨁◯◯ Low^e,h^
Small for gestational age (SGA) [N: 4840; 5 RCTs]	RR 0.83 (0.73 to 0.94)	32.4%	26.6% (24.6 to 28.8)	5.8% fewer (7.8 fewer to 3.6 fewer)	⨁⨁⨁◯ Moderate^f^
Low birth weight (LBW) [N: 4855; 5 RCTs]	RR 0.83 (0.73 to 0.95)	22.3%	18.5% (16.5 to 20.5)	3.8% fewer (5.8 fewer to 1.8 fewer)	⨁⨁⨁◯ Moderate^f^
Maternal anemia [N: 2746; 2 RCTs]	RR 0.96 (0.88 to 1.05)	40.6%	39.4% (35.7 to 43)	1.2% fewer (4.9 fewer to 2.4 more)	⨁⨁⨁◯ Moderate^j^

Patient or population: Non-pregnant women. Setting: Community. Intervention: Multiple micronutrients (MMN). Comparison: Iron–folic acid (IFA). ^*^The risk in the intervention group (and its 95% confidence interval) is based on the assumed risk in the comparison group and the relative effect of the intervention (and its 95% CI). CI, confidence interval; MD, mean difference; RR, risk ratio; RCT, randomized controlled trial. GRADE Working Group grades of evidence. High certainty: We are very confident that the true effect lies close to that of the estimate of the effect. Moderate certainty: We are moderately confident in the effect estimate: the true effect is likely to be close to the estimate of the effect, but there is a possibility that it is substantially different. Low certainty: Our confidence in the effect estimate is limited: the true effect may be substantially different from the estimate of the effect. Very low certainty: We have very little confidence in the effect estimate: the true effect is likely to be substantially different from the estimate of effect. Explanations: ^a^All are open-label trials; ^b^high heterogeneity noted; ^c^trial neither registered nor traceable at the trial registry, and the effect looks implausible; ^d^extreme heterogeneity noted; ^e^95% confidence interval is wide; fall except one are open-label trials; ^g^95% confidence interval is very wide; hhalf of the trials were open-label; ^i^majority are open-label trials; ^j^one trial was open-label, and the other had a high attrition rate.

## Discussion

### Summary of evidence

After an extensive search of the literature, we found 12 studies eligible for inclusion (data of 11,391 participants). For the primary outcomes, there was no significant difference in the birth weight [MD, 35.61 (95% CI, −7.83 to 79.06), *p* = 0.11], birth length [MD, 0.19 (95% CI, −0.03 to 0.42), *p* = 0.09], and head circumference [MD, −0.25 (95% CI, −0.64 to −0.14), *p* = 0.22] between the MMN and control groups. The GRADE evidence generated was of “very low certainty.” For all the secondary outcomes (except for SGA and LBW), the difference between the MMN and control groups was not significant. There was a significant decrease in the rate of SGA and LBW newborns in the MMN group, and the evidence generated was of “moderate certainty.”

In the subgroup analysis, studies providing lipid-based MMN supplementation in sachet form found significant differences in birth weight [MD, 58.77 g] and length [MD, 0.36 cm] between the MMN and control groups. This is in agreement with previously published data. In the Cochrane review (four trials from LMICs, 8,018 pregnant women) published in 2018, the authors found that newborns in the lipid-based MMN group had a slightly higher mean birth weight (MD 53.28 g) and length (MD 0.24 cm) (27). In a recent trial from Pakistan, the authors included 60 underweight women with pre-eclampsia (28). The lipid-based MMN supplementation group had a significantly higher birth weight (mean difference (MD), 121 g) and length (MD, 0.57 cm) compared to the control group.

There have been conflicting results from trials on MMN vs. IFA supplementation started during the preconception period with some showing benefits, whereas others did not. In one trial from Vietnam, there were no significant differences in the growth and development scores in the offspring between the MMN and IFA groups (14). However, another multi-country three-arm trial found that MMN supplementation led to a higher birth length and less number of SGA newborns with a low rate of stunting (16). However in this trial, for unknown reasons, the Guatemala site did not show a significant difference in the above outcomes between the MMN and IFA groups. In another trial from South Asia, the MMN improved birth weight and reduced stunting and wasting in newborns compared to IFA (18). In this trial, a better effect was seen when the supplementation was initiated >3 months before conception. In the follow-up at 24 months of age, there was no significant difference between MMN and standard care groups (19). The reason for finding no difference might have been due to a small sample size resulting from a high loss of follow-up in the study population. In another large trial, there was no difference in the linear growth and cognitive development between offsprings of MMN (supplemented as capsules) and IFA groups at 2-year follow-up (11). The same group of authors did not find any difference in the postpartum depressive score among mothers in either of the groups (14). A trial assessing placental function in MMN and IFA supplemented found no significant difference, and the same effect was found for the fetal and birth outcomes (15). However, a trial from Vietnam has findings that contrast with the above trials in which simple FA supplementation was in no way different (with regard to the birth weight) from either MMN or IFA supplementation started during the preconception period (10). However, the authors could not provide a suitable explanation for the same. A trial from Indonesia found that fetal survival was significantly better in the MMN-supplemented group (12). A trial from Turkey found no difference between IFA- and MMN-supplemented groups in hemoglobin concentration. In a trial from Indonesia, the fetal length was better in those supplemented with MMNs during the preconception period (20).

In the present systematic review, an overall consistent effect of MMN supplementation was not found. A 17% decrease was observed in the rates of LBW and SGA without affecting other neonatal and maternal outcomes. These findings are similar to those of the findings from antenatal supplementation of MMNs, where the reduction was ≥10% (3, 8, 9). This implies that the differences are more pronounced in the group of children born below the limits considered by the WHO standards as low weight/age and low height/age. However, getting a straight plausible answer for these discrepancies is not an easy task. However, possible explanations, as agreed upon by other researchers, include diversities in the composition and supplementation of MMN formulations or influences from effect modifiers (3, 8, 9). The latter could be the age at enrollment [<20 years vs. ≥20 years, parity (primiparous vs. multiparous)], body mass index (BMI) (<18.5 [underweight] vs. ≥18.5 [not underweight]), maternal height (short stature [<150 cm] vs. normal stature [≥150 cm]), maternal education (none vs. some), and maternal anemia (hemoglobin) status at enrollment (<11 g/dL [anemic] vs. ≥11 g/dL [non-anemic]), gestational age at enrollment (<13 weeks vs. ≥13 weeks), and region (Africa vs. Asia) (8).

In an individual patient data meta-analysis including data from trials on antenatal MMN, the authors observed improved survival for female neonates and greater birth-outcome benefits for infants born to malnourished and anemic pregnant women (9). In addition, early initiation in pregnancy and high adherence to the supplements also provided greater overall benefits.

### Limitations

The studies were variable in many aspects (blinding of participants and outcome assessors, type and dose schedule of the supplements, duration of administration, and outcome measurements). Many were open-label studies and had high attrition rates. Of the 12 included studies, 11 were conducted in the Asian population.

### Future areas of research

Good-quality trials need to be designed to answer the research questions related to MMN supplementation in diverse settings. Future studies should be more uniform regarding the type of MMN and their supplements (composition, dose, schedule, and duration of supplementation). More follow-up data on neurodevelopmental outcomes in the offspring should be included. Future studies should also include cost–benefit outcomes. More data should come from other LMICs outside the Asian population.

## Conclusion

A “very low certainty” of evidence suggests that MMN supplementation may not be better than routine IFA supplementation in improving newborn anthropometric parameters (weight, length, and head circumference). The adverse events resulting from the supplementation were not significant. We need better quality uniformly designed RCTs before any firm recommendation can be made.

## Data availability statement

The original contributions presented in the study are included in the article/Supplementary material, further inquiries can be directed to the corresponding author.

## Author contributions

RD: Conceptualization, Data curation, Formal analysis, Funding acquisition, Investigation, Methodology, Project administration, Resources, Software, Supervision, Validation, Visualization, Writing – original draft, Writing – review & editing. JS: Conceptualization, Data curation, Formal analysis, Supervision, Writing – original draft, Writing – review & editing. NJ: Conceptualization, Formal analysis, Methodology, Software, Supervision, Writing – original draft, Writing – review & editing. BD: Conceptualization, Funding acquisition, Methodology, Supervision, Writing – original draft, Writing – review & editing. AS: Formal analysis, Investigation, Methodology, Resources, Writing – original draft, Writing – review & editing. PP: Methodology, Project administration, Resources, Software, Visualization, Writing – original draft, Writing – review & editing. PS: Data curation, Formal analysis, Investigation, Project administration, Resources, Writing – original draft, Writing – review & editing.

## Footnotes

^1^www.clinicaltrials.gov, ISRCTN Registry, www.who.int/ictrp/en
