# Supplementary material for: Effect of preconception multiple micronutrients vs. iron–folic acid supplementation on maternal and birth outcomes among women from developing countries: a systematic review and meta-analysis
Source: Front Nutr. 2024 Jun 14;11:1390661. doi: 10.3389/fnut.2024.1390661 (PMC11211373; doi:10.3389/fnut.2024.1390661)
Supplement: Supplementary file 1 [file Table_1.DOCX]

**Supplementary table 1: Search strategies used for MEDLINE, EMBASE and the Cochrane Central Register of Controlled Trials (CENTRAL)**

| **MEDLINE**   \| 1 \| Pregnancy/ \| \| --- \| --- \| \| 2 \| pregnan*.mp. \| \| 3 \| gravid*.mp. \| \| 4 \| gestation*.mp. \| \| 5 \| Pregnant Women/ \| \| 6 \| prepregnan*.mp. \| \| 7 \| pre-pregnan*.mp. \| \| 8 \| preconception*.mp. \| \| 9 \| (child adj3 bearing).mp. \| \| 10 \| (child* adj3 bear*).mp. \| \| 11 \| matern*.mp. \| \| 12 \| prenatal.mp. \| \| 13 \| pre-natal.mp. \| \| 14 \| perinatal.mp. \| \| 15 \| peri-natal.mp. \| \| 16 \| antenatal.mp. \| \| 17 \| ante-natal.mp. \| \| 18 \| or/1-17 \| \| 19 \| exp Vitamins/ \| \| 20 \| vitamin*.mp. \| \| 21 \| exp Minerals/ \| \| 22 \| mineral*.mp. \| \| 23 \| exp Micronutrients/ \| \| 24 \| micronutrient*.mp. \| \| 25 \| micro-nutrient*.mp. \| \| 26 \| exp Diet/ \| \| 27 \| diet*.mp. \| \| 28 \| nutri*.mp. \| \| 29 \| exp Dietary supplements/ \| \| 30 \| supplement*.mp. \| \| 31 \| or/19-30 \| \| 32 \| "randomized controlled trial".pt. \| \| 33 \| "controlled clinical trial".pt. \| \| 34 \| (random$ or placebo$).tw,sh. \| \| 35 \| ((singl$ or double$ or triple$ or treble$) and (blind$ or mask$)).tw,sh. \| \| 36 \| single-blind method/ \| \| 37 \| double-blind method/ \| \| 38 \| or/32-37 \| \| 39 \| 18 and 31 and 38 \| \| 40 \| (animals not human).mp. \| \| 41 \| 39 not 40 \| |
| --- | --- | --- | --- | --- | --- | --- | --- | --- | --- | --- | --- | --- | --- | --- | --- | --- | --- | --- | --- | --- | --- | --- | --- | --- | --- | --- | --- | --- | --- | --- | --- | --- | --- | --- | --- | --- | --- | --- | --- | --- | --- | --- | --- | --- | --- | --- | --- | --- | --- | --- | --- | --- | --- | --- | --- | --- | --- | --- | --- | --- | --- | --- | --- | --- | --- | --- | --- | --- | --- | --- | --- | --- | --- | --- | --- | --- | --- | --- | --- | --- | --- | --- |
| **EMBASE**   \| 1 \| Pregnancy/ \| \| --- \| --- \| \| 2 \| pregnan*.mp. \| \| 3 \| gravid*.mp. \| \| 4 \| gestation*.mp. \| \| 5 \| Pregnant Women/ \| \| 6 \| prepregnan*.mp. \| \| 7 \| pre-pregnan*.mp. \| \| 8 \| preconception*.mp. \| \| 9 \| (child adj3 bearing).mp. \| \| 10 \| (child* adj3 bear*).mp. \| \| 11 \| matern*.mp. \| \| 12 \| prenatal.mp. \| \| 13 \| pre-natal.mp. \| \| 14 \| perinatal.mp. \| \| 15 \| peri-natal.mp. \| \| 16 \| antenatal.mp. \| \| 17 \| ante-natal.mp. \| \| 18 \| or/1-17 \| \| 19 \| exp Vitamins/ \| \| 20 \| vitamin*.mp. \| \| 21 \| exp Minerals/ \| \| 22 \| mineral*.mp. \| \| 23 \| exp Micronutrients/ \| \| 24 \| micronutrient*.mp. \| \| 25 \| micro-nutrient*.mp. \| \| 26 \| exp Diet/ \| \| 27 \| diet*.mp. \| \| 28 \| nutri*.mp. \| \| 29 \| exp Dietary supplements/ \| \| 30 \| supplement*.mp. \| \| 31 \| or/19-30 \| \| 32 \| Clinical trial/ \| \| 33 \| randomized controlled trial/ \| \| 34 \| controlled clinical trial/ \| \| 35 \| multicenter study/ \| \| 36 \| Phase 3 clinical trial/ \| \| 37 \| Phase 4 clinical trial/ \| \| 38 \| exp RANDOMIZATION \| \| 39 \| Single Blind Procedure/ \| \| 40 \| Double Blind Procedure/ \| \| 41 \| Crossover Procedure/ \| \| 42 \| PLACEBO/ \| \| 43 \| randomi?ed controlled trial$.tw. \| \| 44 \| rct.tw. \| \| 45 \| (random$ adj2 allocat$).tw. \| \| 46 \| single blind$.tw. \| \| 47 \| double blind$.tw. \| \| 48 \| ((treble or triple) adj blind$).tw. \| \| 49 \| placebo$.tw. \| \| 50 \| Prospective Study/ \| \| 51 \| or/32-50 \| \| 52 \| 18 and 31 and 51 \| \| 53 \| (animals not human).mp. \| \| 54 \| 52 not 53 \| |
| **Cochrane Central Register of Controlled Trials (CENTRAL)**   \| 1 \| Pregnan* \| \| --- \| --- \| \| 2 \| MeSH descriptor: [Pregnancy] explode all trees \| \| 3 \| Gestation \| \| 4 \| Pregnant women \| \| 5 \| MeSH descriptor: [Pregnant Women] explode all trees \| \| 6 \| Expect* \| \| 7 \| mother OR matern* \| \| 8 \| gravidity \| \| 9 \| MeSH descriptor: [Gravidity] explode all trees \| \| 10 \| Prepregnan* OR preconception OR periconception \| \| 11 \| Antenatal* OR Prenatal* OR Perinatal* \| \| 12 \| #1 OR #2 OR #3 OR #4 OR #5 OR #6 OR #7 OR #8 OR #9 OR #10 OR #11 \| \| 13 \| Vitamins \| \| 14 \| MeSH descriptor: [Vitamins] explode all trees \| \| 15 \| Micronutrients \| \| 16 \| MeSH descriptor: [Micronutrients] explode all trees \| \| 17 \| Minterals \| \| 18 \| MeSH descriptor: [Minerals] explode all trees \| \| 19 \| Dietary supplements \| \| 20 \| MeSH descriptor: [Dietary Supplements] explode all trees \| \| 21 \| Supplement* \| \| 22 \| Multivitamins \| \| 23 \| #13 OR #14 OR #15 OR #16 OR #17 OR #18 OR #19 OR #20 OR #21 OR #22 \| \| 24 \| MeSH descriptor: [Animals] explode all trees \| \| 25 \| MeSH descriptor: [Humans] explode all trees \| \| 26 \| #24 NOT #25 \| |
