# Supplementary figures and images for: Effect of preconception multiple micronutrients vs. iron–folic acid supplementation on maternal and birth outcomes among women from developing countries: a systematic review and meta-analysis
Source: Front Nutr. 2024 Jun 14;11:1390661. doi: 10.3389/fnut.2024.1390661 (PMC11211373; doi:10.3389/fnut.2024.1390661)

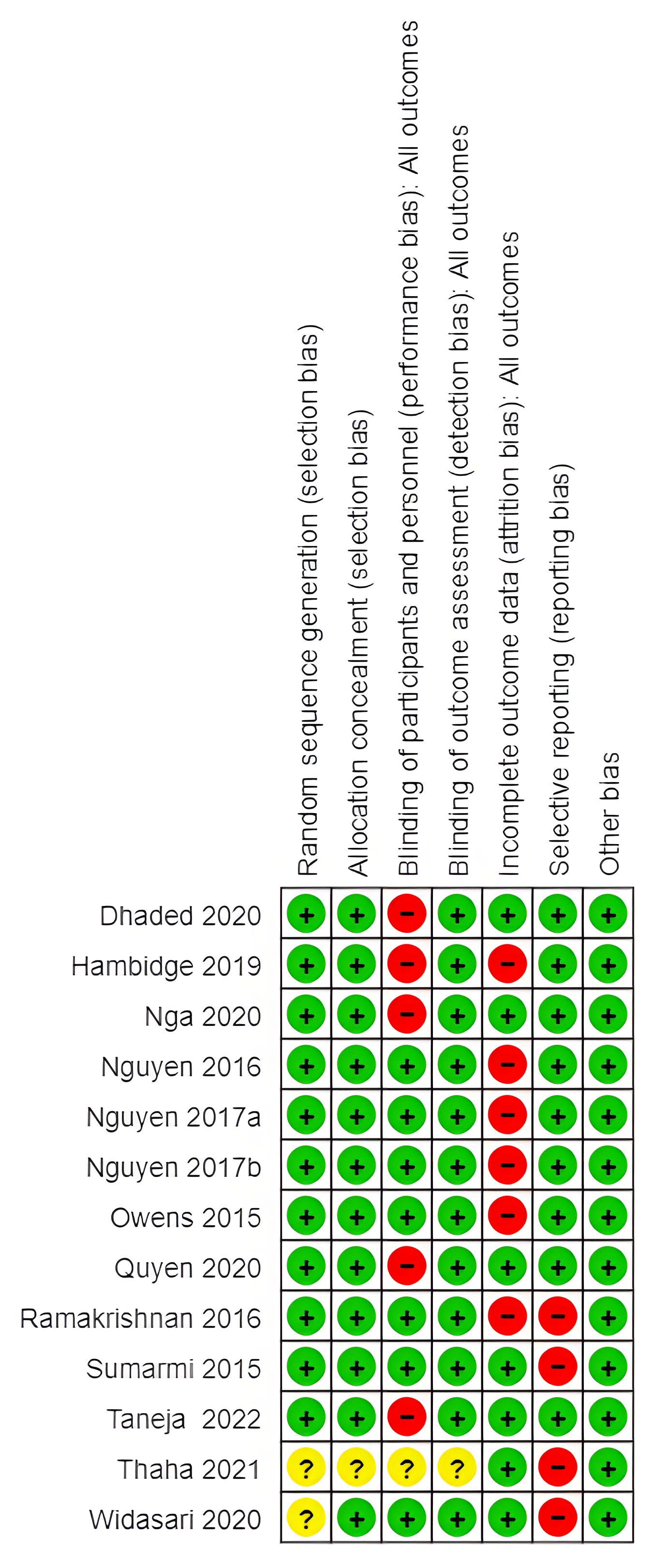

Supplement: Supplementary file 2 [file Image_1.TIFF]

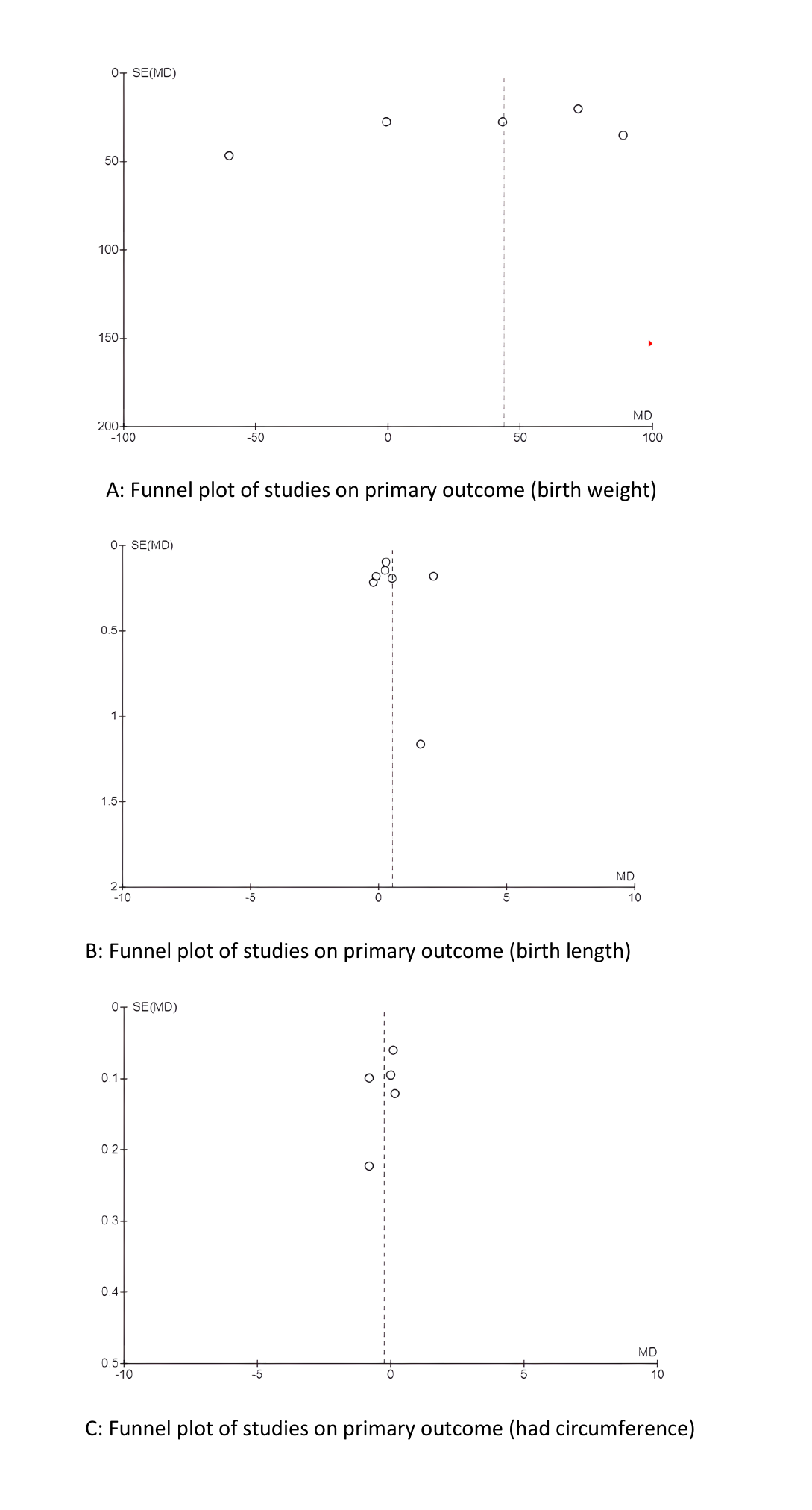

Supplement: Supplementary file 3 [file Image_2.TIFF]
